# Preparation strategy of hydrogel microsphere and its application in skin repair

**DOI:** 10.3389/fbioe.2023.1239183

**Published:** 2023-07-24

**Authors:** Honggang Chi, Yunqi Qiu, Xiaoqing Ye, Jielin Shi, Ziyi Li

**Affiliations:** ^1^ The First Dongguan Affiliated Hospital, Guangdong Medical University, Dongguan, China; ^2^ The Second Clinical Medical College, Guangdong Medical University, Dongguan, China

**Keywords:** hydrogel microsphere, functional microsphere, bioactive microsphere, antibacterial microsphere, hemostatic microsphere, skin repair

## Abstract

In recent years, hydrogel microsphere has attracted much attention due to its great potential in the field of skin repair. This paper reviewed the recent progress in the preparation strategy of hydrogel microsphere and its application in skin repair. In this review, several preparation methods of hydrogel microsphere were summarized in detail. In addition, the related research progress of hydrogel microspheres for skin repair was reviewed, and focused on the application of bioactive microspheres, antibacterial microspheres, hemostatic microspheres, and hydrogel microspheres as delivery platforms (hydrogel microspheres as a microcarrier of drugs, bioactive factors, or cells) in the field of skin repair. Finally, the limitations and future prospects of the development of hydrogel microspheres and its application in the field of skin repair were presented. It is hoped that this review can provide a valuable reference for the development of the preparation strategy of hydrogel microspheres and promote the application of hydrogel microspheres in skin repair.

## 1 Introduction

In recent years, hydrogel microspheres have shown remarkable potential to serve as a key polymer scaffold for tissue repair. The porous structure of hydrogel microspheres holds a variety of functions, such as enhancing the mechanical strength, stability, and ductility of materials, regulating drug release, providing more anchor points for cross-linking with other materials, and solving the shortcomings of hydrogel such as fragility (Zhang et al., 2022; Wang et al., 2023). Several hydrogel microspheres could transmit physical/chemical signals to damaged sites to provide anchor points for the occurrence and continuation of biological reactions. Meanwhile, many studies have shown that bioactive substances (growth factors, cells, etc.) and drugs could be encapsulated in hydrogel microspheres, forming biological or functional materials with good biocompatibility to meet various wound microenvironments (involving inflammation, ischemic, immunology, etc.) and achieve multifunctional effects (Zhang R. et al., 2020; Hamilton et al., 2021; Pan et al., 2021; Yuan et al., 2021; Ji et al., 2022; Lin et al., 2022; Yu et al., 2022; Zhao et al., 2022). In terms of preparation and production, we summarized the research progress in hydrogel microspheres and introduced the preparation strategy including microfluidic, emulsion, photomask, 3D printing, and electrospray ionization and described its advantages and related biomedical applications.

As the largest tissue in the human body, skin plays an important role in protecting the body. It is a natural biological barrier, insulating against viruses, microorganisms, and mechanical and physical factors such as temperature changes. Normally, lighter or superficial skin wounds can repair themselves. But larger and deeper wounds on the skin are not easy to repair. Skin wound dressing is always applied for promoting skin repair (Negut et al., 2018; Obagi et al., 2019; Sinha, 2019; Yao et al., 2021; Su et al., 2022). Although traditional wound dressings (gauze and bandages, etc.) have been widely used, they also hold some disadvantages, such as single performance, poor air permeability, and lack of biodegradability and bioactivity (Naahidi et al., 2017; Farahani and Shafiee, 2021). Hydrogel microspheres as wound dressings have multifunctional properties such as biodegradability, biocompatibility, reactivity, and injectable, and have a wider application prospect (Zhang H. et al., 2021; Peng et al., 2023; Shi et al., 2023; Zhang et al., 2023). Additionally, the structure of hydrogel microspheres endows them with the capacity for promoting cell proliferation and migration and nutrient and waste transportation. Hydrogel microspheres could also serve as a microcarrier platform for delivery of drugs, bioactive factors, or cells to the wound site, accelerating wound healing.

In this paper, we summarized the research progress in hydrogel microspheres and introduced the preparation strategy of hydrogel microspheres including microfluidics, emulsion, photomask, 3D printing, and electrospray ionization. Then, the different functions and detailed mechanisms of hydrogel microspheres in the field of skin repair were summarized. Finally, the development of direction of hydrogel microspheres were discussed. This review is expected to provide a thinking anchor for subsequent research in the preparation and design of hydrogel microspheres and its biomedical application.

## 2 Preparation strategy of hydrogel microsphere

**FIGURE 1:**
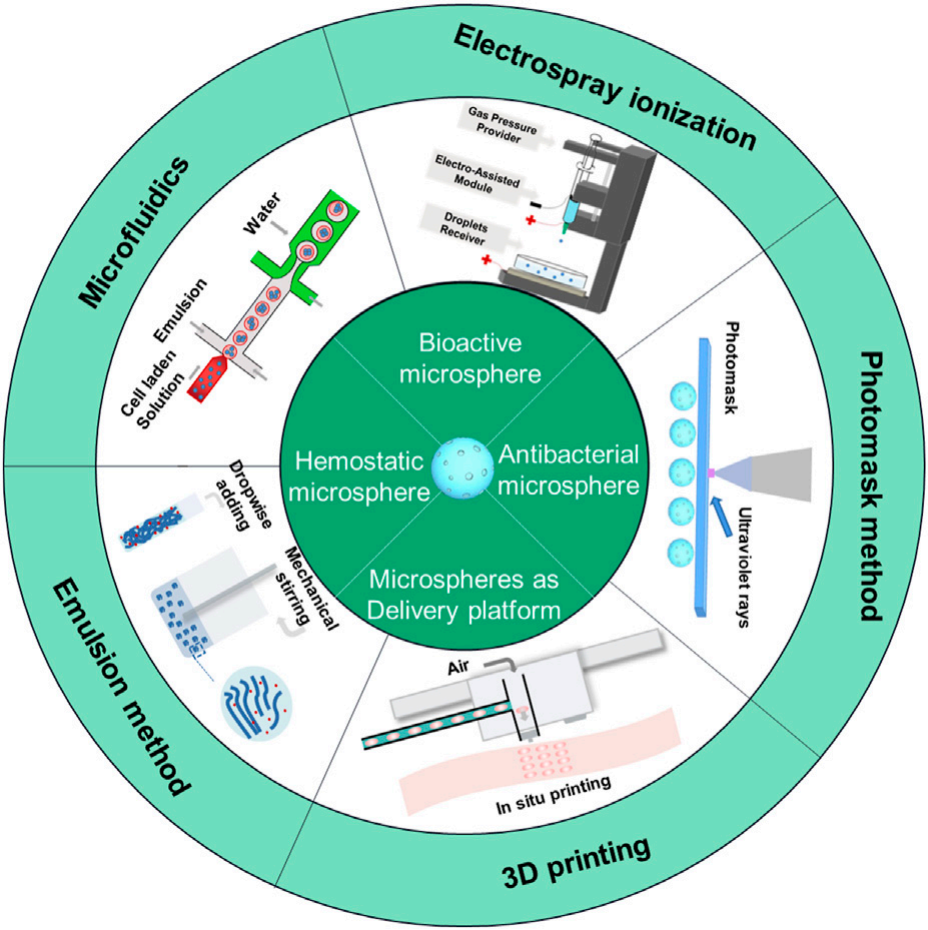
Scheme of preparation method of hydrogel microsphere.

A hydrogel microsphere is a 3D cross-linked network of hydrophilic polymers; they have attracted great attention in drug delivery and biomedical application fields (Zhang R. et al., 2020; Zhang H. et al., 2021; Datta et al., 2021; Zhang Q. et al., 2021; Guan et al., 2021; Yuan et al., 2021). Many studies have reported on the preparation techniques of hydrogel microspheres. From the material point of view, the properties directly depend on the composition, structure, and processing techniques. Therefore, it is necessary to analyze the advantages and disadvantages of various preparation technologies and expand the research and applications. The preparation technology of hydrogel microspheres is still an interesting research field. This chapter provides an overview of several preparation techniques for hydrogel microspheres, including microfluidics, emulsion, photomask, 3D printing, and electrospray ionization (Figure 1). These approaches have greatly facilitated communication and collaboration among multiple disciplines and contributed to the development of novel manufacturing technologies and materials for biomedical applications in tissue engineering and precision medicine.

### 2.1 Microfluidics

In recent years, microfluidic technology was developed to fabricate hydrogel microspheres. With the microfluidic flowing mechanism, the preparation of hydrogel microspheres can be more precisely controlled and microspheres with a uniform size distribution can be obtained. Notably, in many studies, the size and morphology of the prepared microspheres have been shown to have a very profound impact on their application in different fields such as drug sustained release, cell coating, biological detection, etc. Therefore, the preparation of hydrogel microspheres with a narrow range of size distribution and controllable composition and structure has become a research hotspot. As a consequence, hydrogel microspheres have been widely researched in drug delivery and release (Wong et al., 2018), suspension detection, tissue repair, and other fields.

Various methods were devised to prepare hydrogel microspheres with microfluidics. The resultant hydrogel microspheres have many advantages, such as uniform size, stability, syringeability, and excellent biocompatibility. For example, (Jiang et al. (2012) used microfluidics to prepare a novel discrete immune microsphere using monodisperse droplets as a template. Kim et al. (2008) described a method for synthesizing porous photonic microspheres in coaxial flow capillary devices. Zhang et al. (2009) proposed a novel and simple preparation method to prepare HEMA-MMA microspheres with a large pore structure by using oil-in-water (O/W) type droplets as a template and injecting an initiator and pore-causing agent into the oil phase. Shao et al. (2022) designed a user-friendly microfluidic device with a sharp needle by inserting a commercial tapered opening sharp needle into a transparent silicone tube and generated hydrogel microspheres with uniform and adjustable sizes.

**FIGURE 2:**
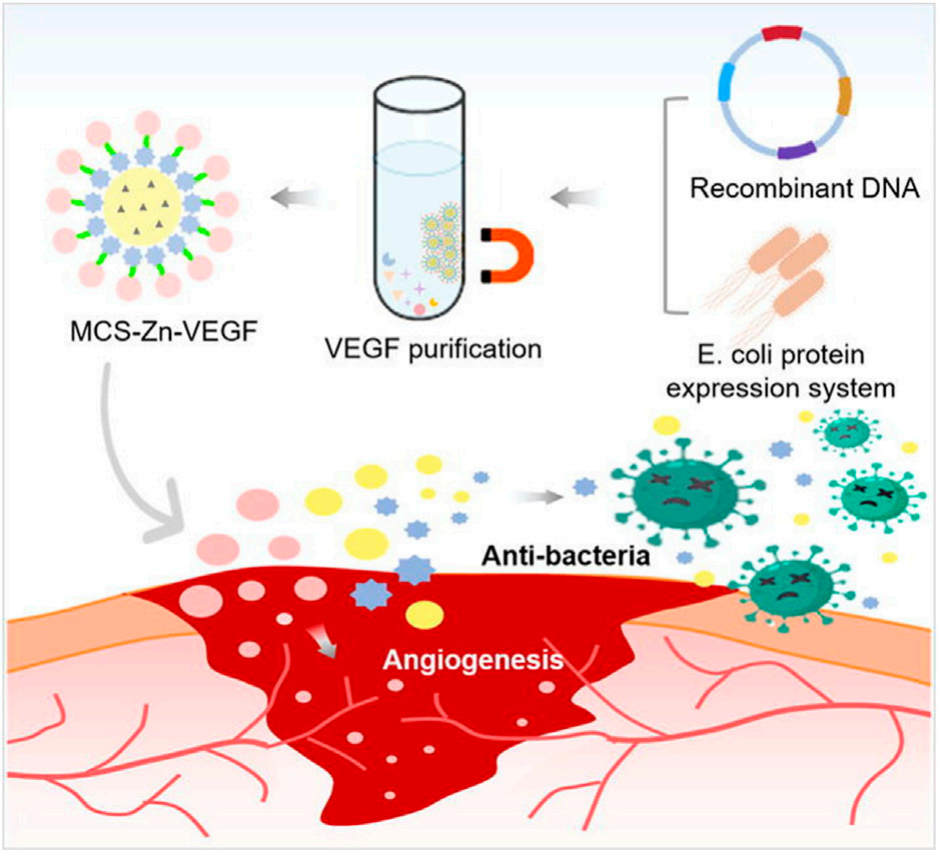
Scheme of fabrication of a multifunctional microsphere and its application in wound repair. The microsphere was fabricated by magnetic chitosan microspheres (MCS) conjugated Zn^2+^ and recombinant VEGF (Lei et al., 2022).

How to fabricate functionalized hydrogel microsphere with a microfluidic technique has also been explored. For instance, Liu et al. (2018) reported a hydrogel microsphere with tunable chemical functionalities toward biomolecular conjugation using a high-throughput double emulsion-based microfluidic method. Shen J. et al. (2022) designed an injectable nano-zyme-functionalized hydrogel microsphere. The microsphere was constructed by grafting manganese dioxide (MnO_2_) -lactate oxidase (LOX) composite nanozyme on microfluidic hyaluronic acid methacrylate microsphere via chemical bonds. Thus, the microspheres could have a long-term oxygen-promoted lactated exhaustive effect and a long half-life *in vivo*. In addition, it could reduce the damage to the enzyme and maximize the maintenance of enzyme activity, promoting local enzymatic concentration and activity enhancement. In addition, Lei et al. (2022) has designed antimicrobial hydrogel microspheres using the microfluidic technique for wound healing (Figure 2). The microfluidic technique could immobilize the Zn^2+^ with hydrogel microspheres, and then fabricate the magnetic chitosan microspheres (MCS). The Zn^2+^ and chitosan in the MCS exhibited a synergetic antibacterial effect towards both Gram-positive and Gram-negative bacteria. Furthermore, the MCS could combine the VEGF via coupling, keeping the bioactivity of VEGF in the MCS as much as possible. Thus, the hydrogel microspheres promoted skin wound healing.

### 2.2 Emulsion method

In the emulsion method, two kinds of insoluble solvent under the action of surfactant was used to form a uniform emulsion and solid phase precipitation from the emulsion so that nucleation, growth, agglomeration, agglomeration, and other processes are limited in small spherical droplets. To form spherical particles, further agglomeration should be avoided between particles. The emulsion method can be subdivided into the emulsion crosslinking method, emulsion polymerization method, and inverse emulsion polymerization method.

The emulsion crosslinking method refers to the method of mixing and stirring two kinds of insoluble solvents to form an emulsion, and then the crosslinking reaction is processed with specific substances to prepare hydrogel microspheres. For example, a hydrogel microsphere was prepared using this method via interpenetrating a polymer network between chitosan and polyacrylamide grafted Guar gel (Kajjari et al., 2011). In the study, acrylamide grafted Guar gum (PAAM-g-GG) was prepared by the emulsion crosslinking method using glutaraldehyde (GA) as a crosslinking agent, and then it was blended with chitosan (CS) to form interpenetrating polymer network (IPN) hydrogel microspheres. In addition, magnetic hydroxyapatite/gelatin microspheres were fabricated by the emulsion cross-linking method. In the study, the antibacterial drugs including tetracycline hydrochloride (TH) and silver sulfadiazine were loaded into the microsphere. Then, composite hydrogels were further prepared and applied in bone repair by compounding hydroxyapatite and the drug-loaded microsphere (Chen et al., 2022). There are several advantages to the hydrogel microspheres obtained by the emulsion crosslinking method. For example, the hydrogel microspheres fabricated by this method always have a spherical structure with smooth surface, high chemical stability, and sufficient space (Gong et al., 2013). In addition, the hydrogel microspheres prepared by this method are suitable for drug transport and precise drug release (Qi et al., 2019).

The emulsion polymerization method involves the monomer reacting with an emulsifier under mechanical stirring. In this method, the monomer is dispersed into water to form emulsion, and then an initiator is added to trigger monomer polymerization. Thermal responsive core-shell hydrogel microspheres were prepared using this method. In the study, the monomers of poly (N-isopropylacrylamide-copolystyrene) [P (NIPAM-co-St)] and poly (N-isopropylacrylamide) (PNIPAM) were stirred to form an emulsion (Gong et al., 2013), and an initiator was added for monomer polymerization. In the process, no surfactant was used. A hydrogel microsphere was prepared based on aqueous polymeric solution of phenol-substituted hyaluronic acid and collagen containing insulin and laccase for the repair of sciatic tissue (Zolfagharzadeh et al., 2023). In general, the emulsion polymerization method has the advantages of fast polymerization speed, high molecular weight, stable dispersion system, easy control, and continuous operation (Xiao et al., 2004).

**FIGURE 3:**
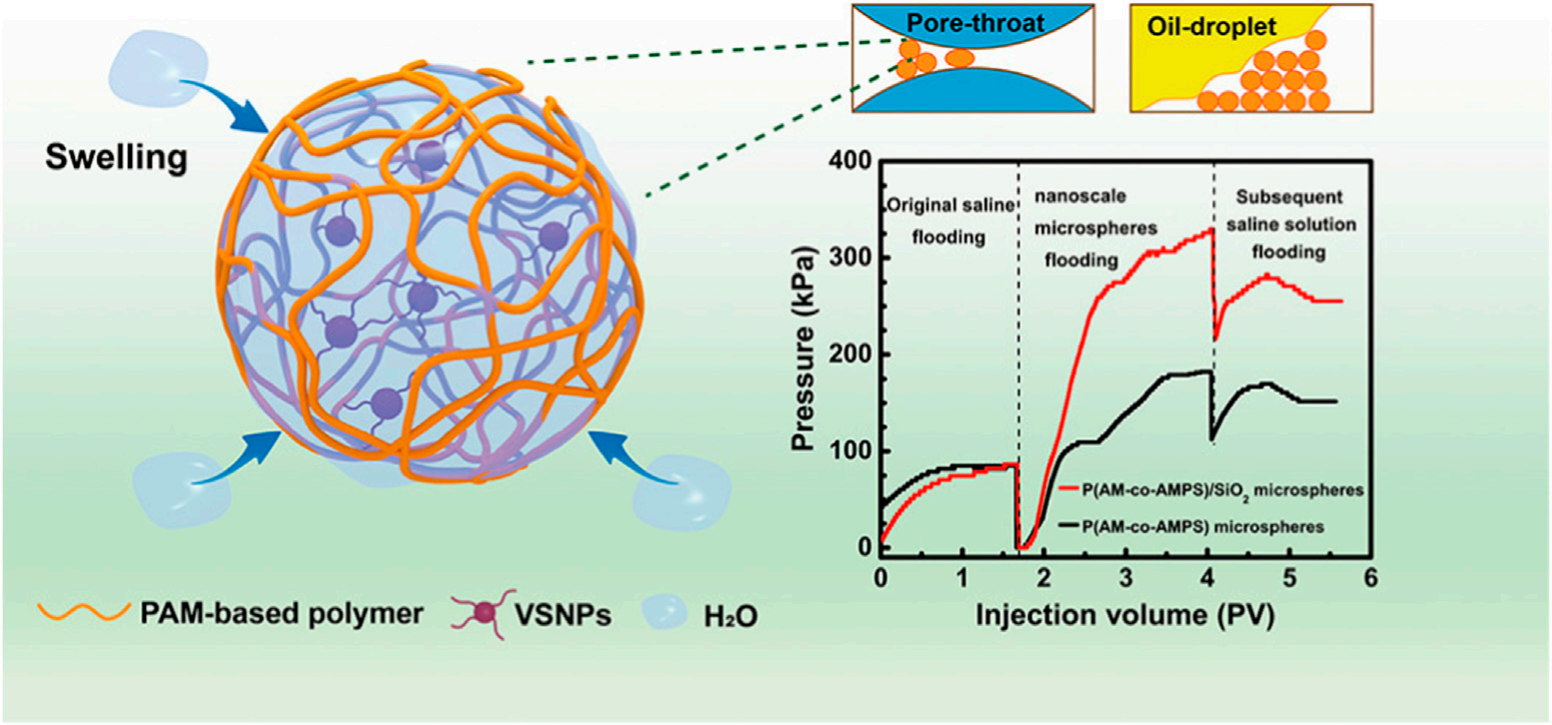
Scheme of the morphology and structure of PAM copolymer hydrogel microspheres by the reversed-phase emulsion polymerization method (Li Z. et al., 2021).

As for the reversed-phase emulsion polymerization method, an aqueous monomer solution prepared by water-soluble monomer is processed to form a water-in-oil emulsion with organic phase under the action of oil-soluble surfactant, and then water-in-oil (water/oil) polymer latex is formed by a polymerization reaction triggered by an oil-soluble initiator (Chang et al., 2020; Liu et al., 2021). Acrylamide (AM) is a monomer commonly used in reversed-phase emulsion polymerization. In the research, PAM copolymer hydrogel microspheres were synthesized by inverted microemulsion copolymerization of acrylamide (AM) and 2-methyl-2-acrylamide propyl sulfonic acid (AMPS) in the presence of vinyl functionalized silica nanoparticles (VSNP) (Figure 3) (Li Z. et al., 2021). Additionally, a porous polyacrylamide hydrogel microsphere was reported to be prepared by inverse emulsion polymerization. The obtained microsphere could be used as a carrier to adsorb lipase by hydrogen bonding interaction (Qin et al., 2022). In short, the reversed-phase emulsion polymerization method has the characteristics of emulsion polymerization and can improve the physical properties of hydrogel microspheres.

### 2.3 Photomask method

Light plays an important role in human society and natural ecology. It is non-contact and controllable in space and can trigger a series of chemical reactions such as photosynthesis. The photomask method is a new light “drilling” technology that uses light as a processing means of soft materials (Yue et al., 2015).

**FIGURE 4:**
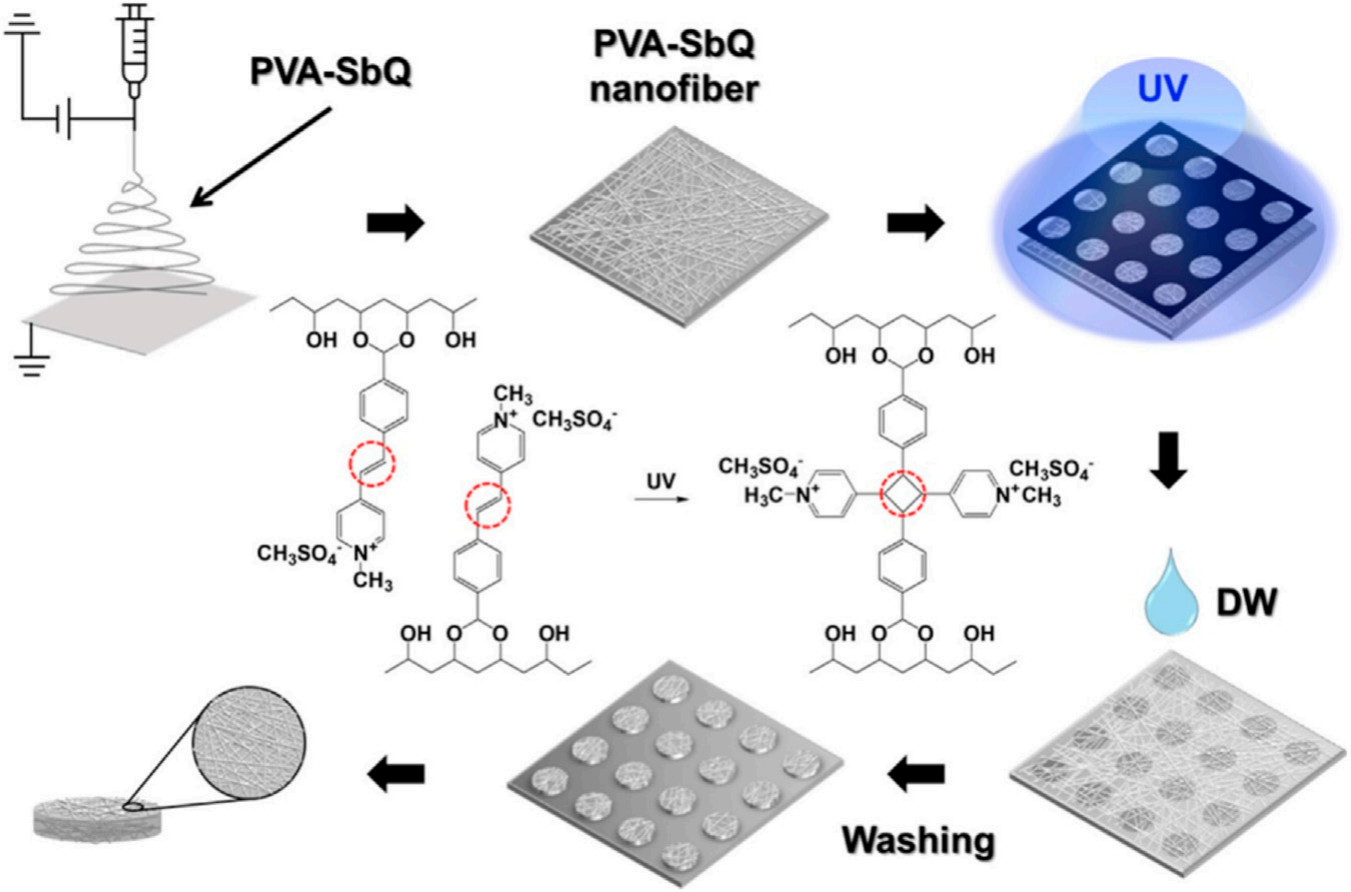
Schematic diagram to illustrate the preparation process of the suspension array of nanofiber microparticles by the combination of electrospinning and photopatterning method (Ham et al., 2023).

The photomask method can also be used to prepare microstructures of hydrogels. Hydrogel microstructures were prepared based on polyethylene (ethylene glycol) diacrylate, dimethacrylate, and tetracrylate, which were patterned photolithographically on a silicon or glass substrate using a photomask (Revzin et al., 2001). The micropattern of hydrogel microspheres can be adjusted by shape and size. In this study, polyaniline (PANi) was added to a polyethylene glycol (PEG) hydrogel matrix using UV-induced photolithography and a mask, and conductive hydrogel micropatterns were generated within seconds (Noh et al., 2021). It was reported that region-controlled porous structures in soft and brittle hydrogels, which resembled jelly, were constructed using the method (Chen et al., 2019). In the study, dynamic crosslinked hydrogels with complex shapes were prepared with the photomask method. Firstly, the experiment was conducted in a frozen state, which created internal mechanical stresses because the resulting ice crystals squeeze the polymer chains (Chen et al., 2019). Then, spatio-temporal UV irradiation in this frozen state led to local stress relaxation through disulfide bond exchange because disulfide crosslinked hydrogels could form a photoinduced network rearrangement. In the final step, subsequent ice melting leads to a porosity pattern, whose porosity could be controlled by light dose. A single patterned sample is used as an adaptive imprint to generate multiple functional devices by exploiting the stimulus responsiveness of hydrogels. With the use of a photomask, various photo defined patterns could be created including square array, flowers, and letters. A hydrogel microsphere was also fabricated by photolithography or the combined method. For example, photolithography combined with liquid crystals promote the synthesis of microparticles with 2D and 3D shapes (Akdeniz and Bukusoglu, 2019). In this study, a photomask was used for photopolymerization of the reactive (RM257). After the unreacted mesogens were extracted, the polymeric microparticles yielded. In addition, electrostatic spinning combined with the photopatterning process generated microparticles based on a photo crosslinkable polymer (Figure 4) (Ham et al., 2023). The design of photomask could control the lateral dimensions and shapes of the microparticles while electrospinning time determined the thickness of the microparticles. The microparticles decorated with silver nanoparticles served as an immunosensor for the detection of biomolecules.

### 2.4 3D printing

3D printing technology is gradually becoming one of the most promising methods for preparing hydrogel microspheres as it can fabricate more accurate and personalized hydrogel microspheres using computer-aided design (CAD) models. 3D printing technology is helpful to mix different materials to construct various microsphere. 3D bioprinting can also effectively integrate bioactive molecules, living cells, and biomaterials to provide open and porous three-dimensional structures for manufacturing biological carriers that are similar to natural biological tissue structures and more functional, making materials more suitable for biomedical applications (Ji et al., 2020; Wen et al., 2022). Compared with other preparation techniques, 3D printing can precisely control the diameter and structure of hydrogel microspheres according to optical and fluid channel parameters, providing a uniform pore distribution. 3D-printed hydrogel microspheres can achieve rapid crosslinking after printing to form uniform texture hydrogel microspheres. And their spherical spatial distribution makes the drug release from all directions of the scaffold, effectively increasing the local drug concentration and reducing the degradation or inactivation of the drug. Additionally, it was demonstrated that the obtained hydrogel microspheres can be used as a higher biomimetic platform to promote the growth of blood vessels and nerves, which is more conducive to accelerating tissue repair and regeneration (Wen et al., 2022). 3D printing technology is often used to manufacture hydrogel microspheres with biological and mechanical properties, which are suitable for cell culture, wound healing, drug delivery, or restoration of clinical tissue and organ function (Park et al., 2019).

**FIGURE 5:**
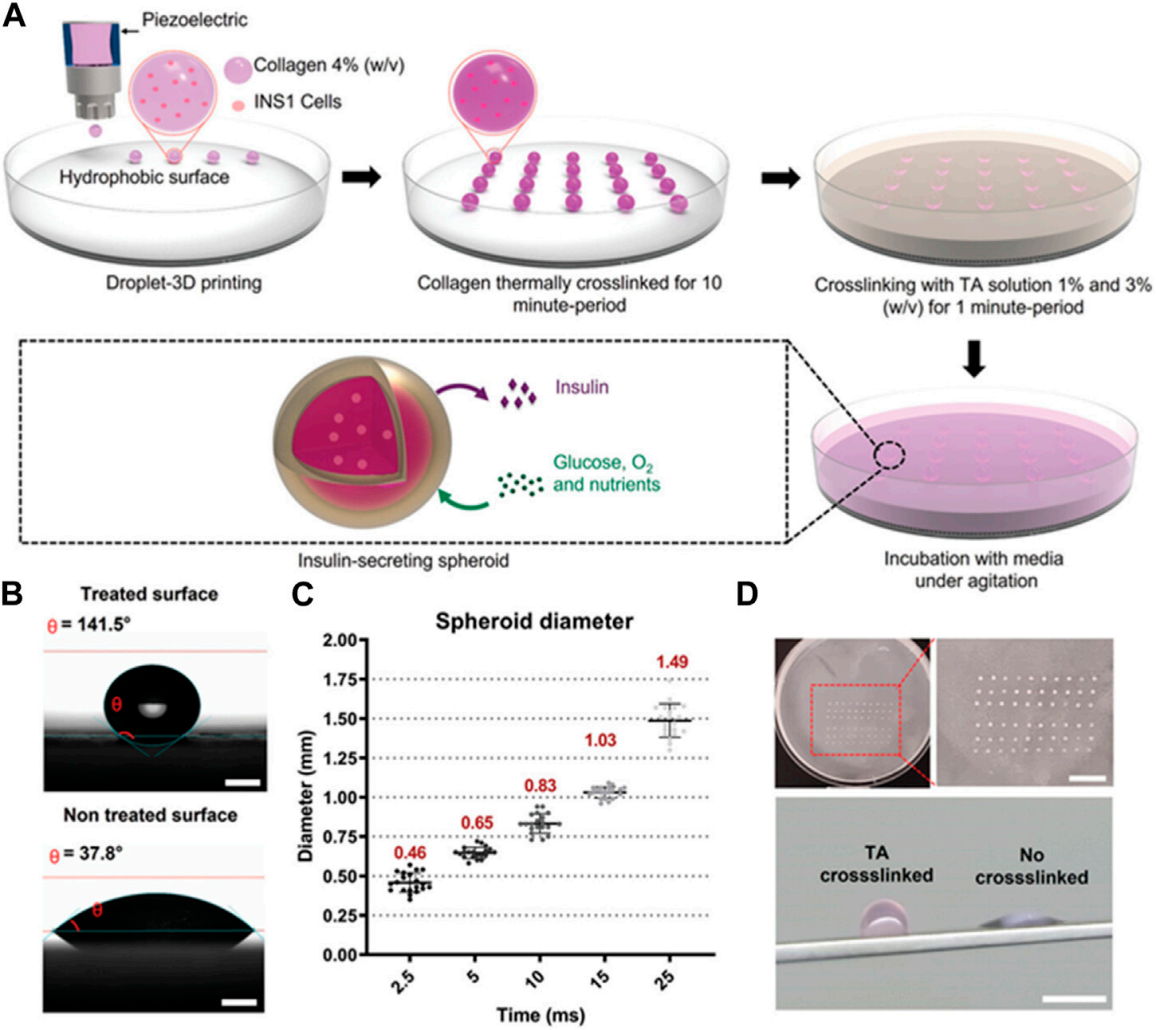
Cell-laden collagen-tannic acid spheroids fabricated by the 3D bioprinter technique. **(A)** Schematic fabrication process of microsphere crosslinked with different concentrations of TA solution. **(B)** Contact angle differences on different substrate and the optical images of the cell-laden spheroids. **(C)** The range of spheroid diameter. The spheroid was generated using the inkjet/valve printhead. **(D)** Images of a square array fabricated using the 3D bioprinter. Scale bar = 10 mm. The images of collagen crosslinked with TA spheroid and collagen control spheroid. Scale bar = 10 mm (Clua-Ferre et al., 2022).

In recent years, 3D printing technology was developed for the preparation of various hydrogel microspheres. To overcome the limitation of current microsphere fabrication technologies such as the need for an oil phase containing surfactants, a 3D bioprinter was employed to prepare a cell-laden collagen microsphere and crosslinked with tannic acid (Figure 5) (Clua-Ferre et al., 2022). The 3D printing approach was developed with a high-throughput methodology for the cell-laden microsphere capable of secreting insulin. 3D printing technology was further united with other strategies to develop hydrogel microspheres. For instance, low-concentration pure GelMA microdroplets were fabricated with the electro-assisted bioprinting method (Xie et al., 2019). The resultant GelMA microdroplets were uniform and the size was about 100 μm. The study suggested that the printed microdroplets had potential in cell therapy, drug delivery, and microspheroidal organoid building. Droplet-based 3D bioprinting has been adopted to precisely generate hydrogel microspheres. The method enables the hydrogel microsphere to form organized structures with high precision. A microfluidic-based printing nozzle was developed to prepare monodispersed microspheres based on high-viscosity bioink. The size of a hydrogel microsphere could be controlled by regulating the flow rates of the fluids. And the obtained hydrogel microsphere could be applied as microcarriers in drug delivery (Zhang S. et al., 2020). Moreover, a 3D-printed device was a key factor to prepare the hydrogel microsphere and tune their size distribution. It has been reported that 3D printing technology was used to prototype a droplet device with controlled screw-and-nut combination and gap height to enable the facile control of the droplet size as well as the droplet generation frequency (Nguyen and Seo, 2022). Ca-alginate microspheres containing A549 cells could be prepared by the proposed 3D printing device. The size of the droplets could be controlled, resulting in the generation of various sizes of cell-encapsulated hydrogel microspheres for drug screening. Additionally, to overcome the low throughput of conventional droplet generators, the three-dimensionally parallelized microfluidic droplet generators were developed (Kamperman et al., 2020). The device was designed with a stacked and radially multiplexed channel. The printed microdevices enable the generation of both solid and core-shell hydrogel microparticles. And it was demonstrated that the cytocompatibility of the hydrogel microparticles as mesenchymal stem cells encapsulated in hydrogel microcapsules could form into stem cell spheroids and remain viable for at least 21 days.

Furthermore, 3D technology has been widely used to construct functionalized hydrogel microspheres or composite scaffolds. For example, Afghah et al. (2020) added different concentrations of silver nitrate to the 3D-printed raw material to improve the antimicrobial performance of the material in the organism. The results showed that the addition of silver nitrate significantly reduced the adhesion of most microorganisms to the scaffold. Wang et al. (2022) mixed methacrylated gelatin (GelMA) with poly (γ-glutamic acid) (PG) microspheres and hydroxy propyl chitosan (CSPO) microspheres to make a compound bioink, which self-assembled to form granular hydrogels with chemical crosslinks through charge interaction between microspheres. It proved that the composite hydrogels combined with the same mass content of PG microspheres and CSPO microspheres displayed good storage modulus, shear thinning ability, and self-healing ability. Meanwhile, this composite bioink also exhibited the potential to enhance the strength of 3D cell printing. Although great progress has been made in the preparation of hydrogel microspheres by 3D printing, the method needs further development in some aspects, such as various equipment parameters, printing environment optimization, and biomolecular activity maintenance.

### 2.5 Electrospray ionization

Electrospray ionization (ESI) is a potential-driven liquid atomization process. It is not only widely used in the field of analytical chemistry as an ionization technique for biomolecular mass spectrometry analysis, but also can be used to prepare hydrogel microspheres via electrostatic forces and ionic crosslinkers. The principle of preparation of hydrogel microspheres by ESI is that the pre-mixed solution is sprayed through a nozzle of the syringe pump and a high voltage is applied at the outlet of the nozzle. In the method, the nozzle of the syringe pump is connected to the positive electrode and the collection device is connected to the negative electrode. Under the combined action of gravity, electric field force, and electrostatic repulsion force, the liquid droplets flowing out of the nozzle gradually form a Taylor cone, and atomize into tiny electriferous droplets. Then, the droplets drop into a solid receiving device (metal collecting plate) or conductive liquid receiving device, and immediately react with other substances to form nanoparticles. ESI can also generate smaller particles through strong shear force and strong voltage, which can be transformed into gas phase ions to prepare aerosols. At present, there are mainly two explanations for the mechanism of the generation of gas phase ions from charged droplets: the ion evaporation model and charge residue model. This spraying process based on the Taylor cone can achieve very fine liquid dispersion (Patriarca et al., 2020).

Compared with other preparation methods, ESI only requires simple instruments including an emitter, a counter electrode, a power supply, a syringe pump for supply of injected liquid, and a collection device (Li J. et al., 2021). Compared to microfluidic methods, ESI is more convenient, simple, and efficient, and it does not require the addition of surfactants, effectively avoiding the complex cleaning process during preparation. Compared with double emulsion, ESI can efficiently prepare hydrogel microspheres with uniform particle size from several microns to thousands of microns by adjusting the parameter control of the system, and has good drug encapsulation efficiency and higher biocompatibility (Li J. et al., 2021). As a consequence, ESI can well protect biomacromolecules whose structures are easily destroyed during dissociation and ionization. ESI has the potential to generate multi-charged ions under specific voltage conditions, so it can be used as a powerful tool to tightly control the deposition of various components, allowing materials to react into hydrogel microsphere structures that can be used to produce biomedical composites. These systems include homogeneous films, arrays, 3D microstructures, packaging systems, and nanofibers (Liu et al., 2020; Tycova et al., 2021).

Although ESI has become an effective method for preparing hydrogel microspheres with uniform particle size and high drug loading rate, it is still necessary to adjust the influencing factors to make the prepared microspheres with a more accurate diameter, structure, and mechanical strength (Tycova et al., 2021). The influence factors include electric field characteristics (external electric field strength, distance, and electrode shape), material properties (surface tension, conductivity, colloidal viscosity of the mixture), and flow rate of the injection pump. In order to meet the demand for application of the ESI method, response surface analysis (RSM) is commonly used to optimize the above experimental parameters and adjust the mixture ratio. RSM can be used to establish and analyze models among several input variables and response variables. Thus, subsequent designs or experiments can be guided by limited experiments, greatly reducing cost and time. According to the experimental analysis, increasing the voltage intensity and surface tension of the mixture will reduce the size of the generated hydrogel microspheres while increasing the flow rate of the injection pump and the viscosity of the mixture will reduce the diameter of the microspheres (Radaei et al., 2017). These findings broaden the possibility for further refinement of material preparation.

## 3 Hydrogel microsphere for skin repair

When skin is damaged, its healing is an extremely complex and dynamic process that can be divided into three main stages: inflammation, proliferation, and tissue remodeling (Shiekh et al., 2020; Matoori et al., 2021; York, 2021). When skin suffers a serious wound, it will not be healed completely for a long time, and can even relapse due to some problems (Shaydakov et al., 2022). Therefore, the development of biomedical materials for wound treatment has important significance in research and clinical application. Although traditional wound dressings (such as gauze, sponges, and bandages) are widely used in clinical practice, their application is also limited due to some disadvantages such as non-degradability and lack of bioactivity (Long C. et al., 2021; Mariani et al., 2021; Truskewycz et al., 2021; Li et al., 2022).

**FIGURE 6:**
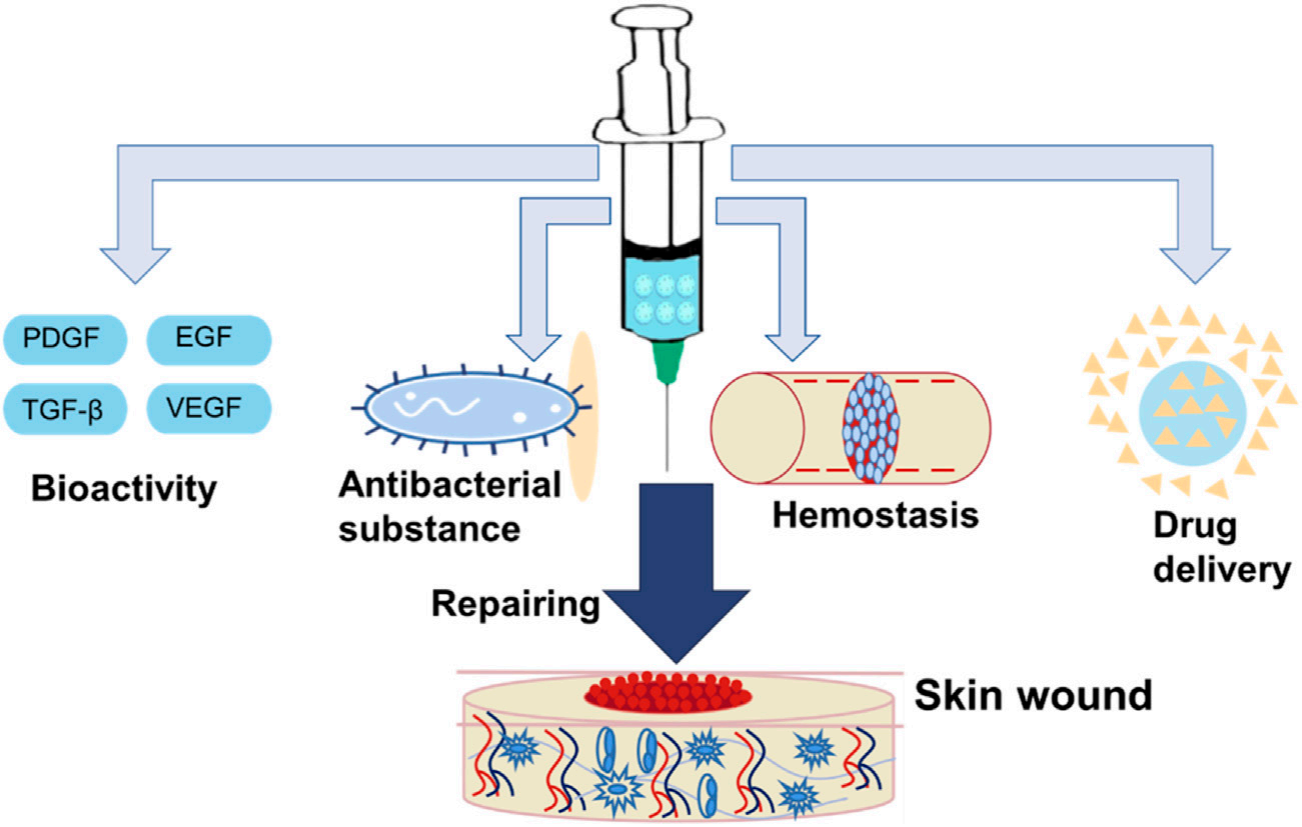
Functional hydrogel microsphere was applied for skin wound healing. It includes bioactive hydrogel microsphere, hemostatic hydrogel microsphere, antibacterial hydrogel microsphere, and hydrogel microsphere as a delivery platform.

As wound dressing, the hydrogel microspheres have attracted attention due to their similar structure to the extracellular matrix (ECM), which can keep the wound moist and absorb excess tissue exudation (Gupta et al., 2020; Liang et al., 2021; Shen J. F. et al., 2022; Wei Q. et al., 2022). In addition, certain functional hydrogel microspheres have excellent biocompatibility and anti-inflammatory activity (Li et al., 2019; Wang et al., 2022). Additionally, the hydrogel microspheres have high porosity, which is beneficial for cell adhesion, cell proliferation, and transport of nutrient and metabolic waste. Therefore, it is a good cell scaffold for wound repair. Meanwhile, the hydrogel microcarrier as a delivery platform to deliver growth factors and drugs for skin repair has aroused the strong interest of researchers. In recent years, functional hydrogel microspheres for skin repair have progressed significantly due to their practicability for wound healing. In the review, we summarized functional hydrogel microspheres for skin repair including bioactive hydrogel microspheres, hemostatic hydrogel microspheres, antibacterial hydrogel microspheres and microspheres to act as a delivery platform (Figure 6).

### 3.1 Bioactive microsphere

Wound dressings prepared from hydrogel microspheres show great potential in the field of skin wound healing. However, during the application process, they still experience a series of problems, such as poor biodegradation and bioactivity, and high storage conditions. Therefore, researchers modify materials by grafting various polar groups and other functional polymers on the surface of biomaterials or polymers to endow them with cell adhesion functions, realizing applications in biomedicine and tissue engineering (Guo et al., 2022). Many corresponding strategies have been adopted, such as modifying the hydrogel microspheres with pro-adhesion groups or grafting the bioactive polymers or bonding bioactive factors.

Schiff base reaction, click reaction, and amidation reaction are popular methods for preparing bioactive hydrogel microspheres. The process of crosslinking reactions and generation of the hydrogel microspheres is driven by chemical reactions. On the one hand, the formed hydrogel microspheres are generally stable and can degrade slowly. On the other hand, the preparation process of the hydrogel microsphere may need suitable reaction conditions and the presence of crosslinkers. Therefore, it should be considered whether reaction conditions and crosslinkers will result in cytotoxicity or immunogenic effects. Besides, bioactive hydrogel microspheres can be fabricated with physical crosslinking methods such as electrostatic interaction, hydrophobic interaction, hydrogen bonding, or other methods (Guo et al., 2022). The method is simple and practicable.

In order to promote skin repair, bioactive and porous hydrogel microspheres were built to mimic the network topology of the skin’s extracellular matrix (ECM), which are essential for promoting cell migration and proliferation, substance transport, congregating coagulation factors, and angiogenesis in damaged skin areas. A composite scaffold with hydrogel microsphere is also vital in wound healing. Liao et al. (2018) developed an injectable composite hydrogel via integration PCEC porous microspheres with the hydrogel crosslinked by calcium gluconate and alginate, which provided more anchor points for fibroblast attachment and promoted skin repair. Celie et al. (2019) designed a composite collagen hydrogel microsphere scaffold by wrapping 1% type I collagen microspheres in 0.3% collagen volume in an appropriate proportion. By optimizing the pore distribution of spheroids, increasing anchor points within the scaffold, and increasing gas transmission rate, cells and blood vessels in damaged skin are effectively induced to migrate and regenerate.

### 3.2 Antibacterial microsphere

It is hard for damaged skin to prevent harmful bacteria from invading tissue, leading to wound infections and serious tissue damage (Ming et al., 2021). Antibacterial hydrogel microspheres play an irreplaceable role in wound healing, especially in the infected wound (Prasad and Gupta, 2020). Hydrogel microspheres have gained researchers’ attention since the larger surface area for ultra-high antimicrobial drug storage and high resistance in bacterial cells when they cluster together. In addition, cationic polymers such as quaternary ammonium compounds (QACs) are popular for the preparation of antibacterial hydrogel microspheres because the polymer has native antibacterial capacity via destroying bacterial cell walls (Saverina et al., 2023).

Researchers developed a composited hydrogel system with catechol-functionalized chitosan/active peptide hydrogel microspheres for skin wound healing (Zhang D. et al., 2021a). CS-C/OPM/β-GP thermosensitive hydrogels were prepared using catechol functionalized chitosan (CS-C) as a polymer matrix, 3.9 μm active oyster peptide microspheres (OPM) as filler, and β-sodium glycerophosphate (β-GP) as a thermal sensitizer. Demonstrated in a skin wound in rats, the hydrogel composed of microspheres not only inhibited the aggregation of various inflammatory cells but also enhanced the synthesis of total protein (TP) in granulation tissue and upregulated the expression of Ki-67 and VEGF in the injury.

Hydrogel microspheres can also serve as a drug platform for the delivery of antibacterial reagents. For example, the antimicrobial hydrogel microspheres loaded with furazolidone (NFZ) and lidocaine (LD) were prepared (Khan et al., 2022). The hydrogel microspheres exhibited negligible damage to cells and accelerated the skin repair of a variety of acute and chronic wounds. In addition, polycyclic triphosphonitrile-co-4,4′-diaminodiphenyl ether (PPO) hydrogel microspheres were prepared by precipitation polymerization using HCCP and 4,4′-diaminodiphenyl ether (ODA) as monomers (Long H. et al., 2021). Silver ion (Ag^+^)-loaded PPO (PPOA) hydrogel microspheres were prepared by *in-situ* load silver nanoparticles onto the surface of hydrogel microsphere. Compared with citric acid modified silver nanoparticles (c-Ag^+^), PPOA hydrogel microspheres showed lower cytotoxicity and good slow releasing performance. The PPOA hydrogel microspheres showed good thermal stability and antibacterial activity against *Escherichia coli* and *Staphylococcus aureus*, promoting wound healing.

### 3.3 Hemostatic microsphere

In the process of skin tissue healing, hemostasis is the first problem to be faced, which includes the dynamic process of coagulation and vascular repair and regeneration. However, the traditional hemostatic materials such as bandages, gels, sponges, adhesives, and powders are all simple in function (Fan et al., 2021; He et al., 2021; Meng et al., 2021; Wang et al., 2021), and may even cause secondary damage to the wound (Kumar et al., 2019). Hemostatic hydrogel microsphere has the advantages of good biocompatibility (Annamalai et al., 2018; Bian et al., 2021; Zhao et al. (2020), slow-release property (Luo et al., 2018), and small size (Sheikhi et al., 2019). In addition, it can act as an excellent microcarrier for hemostatic repairing, which has aroused the interest of researchers. Hence, the development of novel hemostatic material has far-reaching significance.

In recent years, various hemostatic and bioactive hydrogel microspheres were developed. Huang et al. (2021) researched the silk fibroin/alginate hydrogel microspheres with imperforate and rough surface, which were prepared by emulsion cross-linking of sodium alginate (SA) and silk fibroin (SF). The rough surface of hydrogel microspheres could slow down the blood flow rate, contributing to accelerating the coagulation rate. And the porous structure is helpful to absorb blood and avoid the loss of platelets, which is conducive to blood coagulation to a certain extent. Moreover, the hydrogel microspheres were obtained by cross-linking of SA and SF. SF was beneficial to cell adhesion and growth on the surface. Therefore, they claimed that the hydrogel microspheres were biocompatible and could promote cell proliferation.

Additionally, once the tissue was damaged, it is susceptible to suffering inflammatory invasion. Therefore, many hemostatic and anti-inflammatory hydrogel microspheres were designed. Ouyang et al. (2022) reported the cellulose nanocrystal/calcium alginate-based porous microspheres (SA/CNC). The porous microspheres were prepared using an inverse emulsion method (Ouyang et al., 2022). The cellulose nanocellulars (CNC) from different sources (bacteria, algae, and wood) are highly biocompatible, and have shown to be useful in wound healing. And sodium alginate (SA) is a hemostatic material with multi-functions. The hydrogel microspheres prepared by the reverse-emulsion method have shown good coagulation ability and biocompatibility. Besides, the microspheres were negatively charged. Thus, positive charged ε-polylysine (EPL) could be loaded on the hydrogel microspheres by electrostatic interaction, which endowed the hydrogel microspheres with antibacterial properties while stopping bleeding and repairing blood vessels.

Furthermore, composite hemostatic hydrogel microspheres were fabricated for skin repair. Huang et al. (2018) have reported a composite hydrogel microsphere with hemostatic capacity for wound healing. The researchers reported that they combined the hydrogel with PLGA microspheres to achieve sequential drug delivery. In addition, the VEGF encapsulated in PLGA microspheres promotes cell proliferation and differentiation of vascular endothelial cells, accelerating hemostasis and vascular repair in the wound site. Vancomycin was loaded onto the composite system through a reversible Schiff-based reaction to increase the antibacterial effect of the composite system. The hemostatic microspheres prepared by this method can promote angiogenesis and reduce inflammation. They claimed that they provided a new method and means for the preparation of hemostatic microspheres.

### 3.4 Microspheres as a delivery platform

After the skin is injured, many factors including the fibroblast growth factor (FGF) (Bock et al., 2021; Wu et al., 2021; Wei L. et al., 2022), vascular endothelial growth factor (VEGF) (Park et al., 2018; Page et al., 2019; Liu et al., 2022; Xie et al., 2022), and transforming growth factor β (TGF-β) (Xu et al., 2020; Zhang J. et al., 2021; Moreau et al., 2021) are secreted to the areas of injury for angiogenesis and re-epithelization. Some drugs, however, can promote skin repair. Hydrogel microspheres have the advantages of biocompatibility, high porosity, and small volume. Therefore, they have great potential to be used as a delivery platform to carry drugs and active factors for skin repair.

Composite systems via hydrogel combined with microspheres have played an important role in the design of delivery platforms. Xu et al. (2022) have researched a composite system of hydrogel and microspheres. In the study, the CMSS microspheres containing antibacterial drugs were prepared by emulsion crosslinking method. Then, it was coated with carboxymethyl chitosan (CMCS) and carboxymethyl cellulose (CMC) cross-linked hydrogel to obtain the composite system of hydrogel and microsphere. They claimed the prepared dressings have no cytotoxicity and are not irritating to the cells because no toxic chemical crosslinking were utilized in the system. Tetracycline hydrochloride (TH) was loaded in CMCS microspheres. Thus, it not only maintained the antibacterial effect but also avoided the damage to human cells caused by high concentrations of the drug. After it was used to repair wounds, it showed a slow release of the drug and antibacterial effects to protect the wound from infection. In addition, Zhang D. et al. (2021) reported they made a composite system of hydrogel and microspheres. In the system, the catechol-functionalized chitosan (CS-C) was used as a polymer matrix, integral active oyster peptide microspheres (OPM) were used as filler, and β-glycerophospholide sodium (β-GP) was used as a heat sensitizer. They were composited to prepare a composite system of hydrogel and microspheres (CS-C/OPM/β-GP). The hydrogel dressings displayed adhesive capacity to bond the defected tissue together and prevent bacterial infection, facilitating wound healing. Liao et al. (2018) have reported an injectable composite system of hydrogel and microspheres. In the research, the microspheres prepared by poly (ε-caprolactone)-b-poly (ethyleneglycol)-b-poly (ε-caprolactone) (PCL-PEG-PCL, abbreviated as PCEC) have a rough surface, which is conducive to cell attachment and growth. In addition, the microspheres have good stability and biocompatibility. They made the composite system by adding alginate solutions to porous microsphere containing calcium gluconate. It was demonstrated that the composite system has good repair performance as wound dressing via *in vitro* experiments.

**FIGURE 7:**
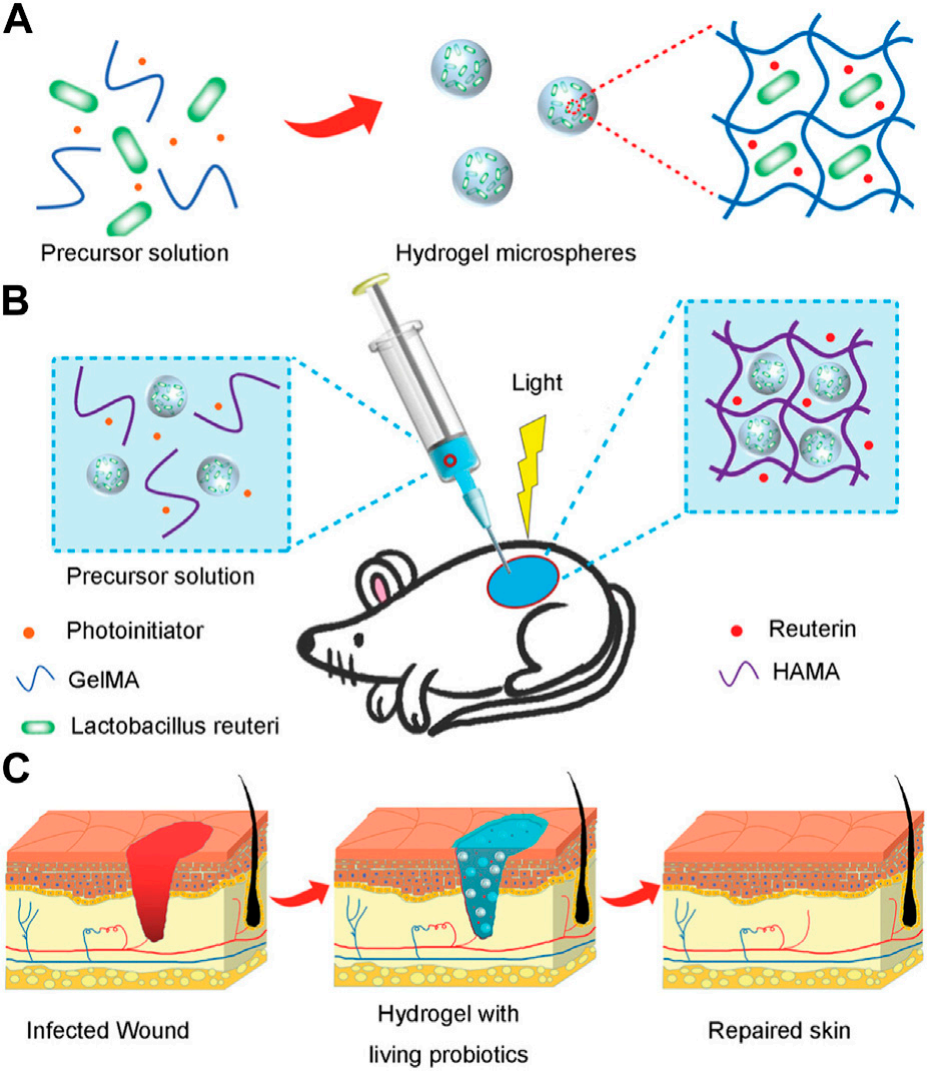
Scheme of composited hydrogels with the living probiotics encapsulated hydrogel microspheres and the process of accelerating infected wound healing. **(A)** Schematic fabrication of living bacteria encapsulated microspheres. **(B)** The gelation *in situ* of living probiotics encapsulated microspheres and HAMA solution by covalent crosslinking under irradiation of light. **(C)** The process of wound healing with treatment of living bacteria hydrogel (Ming et al., 2021).

Functional and bioactive hydrogel microspheres have also been developed for the delivery of drugs or bioactive factors, allowing the delivery of certain substances, including nutrients, biomolecules, lactic acid, and antimicrobial agents. Ming et al. (2021) reported that hydrogel microspheres can be loaded with living probiotics (Figure 7). They presented a novel hydrogel microsphere by encapsulating live bacteria for treating bacterial infections and accelerating wound healing. In the study, the hydrogel microspheres were fabricated by emulsion polymerization, and the L. reuteri was encapsulated in the hydrogel microsphere. Then, under light irradiation, a hydrogel dressing composited with the functional hydrogel microsphere was formed at the wound site by covalent cross-linking of methacrylate modified hyaluronic acid. The resultant hydrogel dressing exhibited less inflammation and accelerated wound repair.

## 4 Conclusion and prospects

In conclusion, hydrogel microspheres have broad potential in repairing skin damage. In the paper, the preparation techniques of microsphere hydrogels and the latest progress in skin repair were described in detail. A series of preparation methods, such as microfluidics, emulsion, photomask, 3D printing, and electrospray ionization, that have been used individually or in combination for the production of multifunctional hydrogel microspheres, were reviewed. As for hydrogel microspheres in the wound healing field, we summarized the functional hydrogel microspheres such as bioactive hydrogel microsphere, antibacterial hydrogel microsphere, hemostatic hydrogel microsphere, and hydrogel microspheres as a delivery platform for skin repair. It is hoped this paper will provide some reference value for the follow-up development of hydrogel microspheres and practical application in skin repair.

Researchers have developed many useful strategies to fabricate novel hydrogel microspheres to broaden the application of hydrogel microspheres as clinical wound dressings. However, more convenient and efficient methods to prepare hydrogel microspheres with uniform size still need to be developed. At the same time, the precise control of the 3D structure and geometric characteristics of hydrogel microspheres also needs to be further improved. Although great progress has been made in the research of physical, chemical, and biological properties of hydrogel microspheres, moreattention should be paid to smart hydrogel microspheres with sensitivity to environmental stimulation by organically combining different materials and changing the preparation or cross-linking between materials. Additionally, the different damage degrees of the wound and the complex microenvironments pose more challenges to the biological properties of the dressing. Hence, the current single functional hydrogel microspheres are not enough to meet these challenges. As a skin wound dressing, hydrogel microspheres need to have more diverse functions. Therefore, the design and preparation of hydrogel microspheres for wound dressings should be considered in a comprehensive way, including but not limited to multifunction, material stability, cytotoxicity, machinability, and enhancement of wound healing. In view of better clinical applications, researchers should aim to develop related devices and new strategies reducing production costs and simplifying preparation methods of hydrogel microspheres.
